# Supplementary material for: Key elements of a successful integrated community-based approach aimed at reducing socioeconomic health inequalities in the Netherlands: A qualitative study
Source: PLoS One. 2020 Oct 20;15(10):e0240757. doi: 10.1371/journal.pone.0240757 (PMC7575081; doi:10.1371/journal.pone.0240757)
Supplement: S1 File — (DOCX) [file pone.0240757.s001.docx]

**S1 File. Consolidated criteria for reporting qualitative studies. COREQ 32-item checklist**

| **No. Item** | **Guide questions/description** | **In our study:** |
| --- | --- | --- |
| **Domain 1: Research team and reﬂexivity** | |  |
| *Personal Characteristics* | |  |
| 1. Interviewer/facilitator | Which authors conducted the interview? | LW |
| 2. Credentials | What were the researcher’s credentials? | LW: MSc, IB: dr. ir., AJS: prof. dr. ir., JCS: prof. dr. ir., CMR: dr. |
| 3. Occupation | What was their occupation at the time of the study? | LW: PhD candidate. IB: associate professor. AJS: professor and dean. JCS: professor. CMR: associate professor. |
| 4. Gender | Was the researcher male or female? | Female: LW, IB, AJS and CMR. Male: JCS. |
| 5. Experience and training | What experience or training did the researcher have? | LW: Interdisciplinary social sciences (MSc) and governance studies (MSc).  Research team: the researchers have backgrounds in health sciences, epidemiology, nutritional sciences and linking policy-practice-research-education on healthy environmental issues. All have experience in research investigating integrated approaches for tackling overweight and stimulating healthy behavior. |
| *Relationship with participants* | |  |
| 6. Relationship established | Was a relationship established prior to study commencement? | LW was introduced to (a part of) the participants by IB, who knew them as being part of the program group. |
| 7. Participant knowledge of the interviewer | What did the participants know about the researcher? e.g. personal goals, reasons for doing the research | The participants knew that these interviews are part of a larger study on the Zwolle Healthy City approach. Both the invitation by mail, the written informed consent and the introduction during the interview mentioned that the study is part of a PhD project. |
| 8. Interviewer characteristics | What characteristics were reported about the interviewer/facilitator? e.g. Bias, assumptions, reasons and interests in the research topic | There are no characteristics reported. The interviewer (LW) was not familiar with the Zwolle Healthy City approach at the start of the research. |
| **Domain 2: study design** | |  |
| *Theoretical framework* | |  |
| 9. Methodological orientation and Theory | What methodological orientation was stated to underpin the study? e.g. grounded theory, discourse analysis, ethnography, phenomenology, content analysis | Thematical content analysis |
| *Participant selection* |  |  |
| 10. Sampling | How were participants selected? e.g. purposive, convenience, consecutive, snowball | Participants were purposively sampled to create a heterogenous interview sample. Purposive sampling is a goal-oriented sampling methods and means that the participants are chosen by the judgment of the researchers LW (PhD candidate) and IB (associate professor). Characteristics on which participants were selected are the organization they work for, the occupation level and their contribution to the implementation of the Zwolle Healthy City approach in the years 2010-2018.  We started to recruit participants at the tactic level of involved organizations by inviting all members of the programme group. The programme group consists of professionals working for the organizations involved as manager, policy adviser or (associate) professor and meets monthly to share and discuss the implementation of the approach.  To collect a wider perspective on the implementation, additional professionals were sampled by asking the programme group members which professionals were involved in the Zwolle Healthy City approach at the operational level.  Additionally, municipal executive councilors who have been involved in the Zwolle Healthy City approach since the start in 2010 and work at the strategic level, were asked to participate in an interview |
| 11. Method of approach | How were participants approached? e.g. face-to-face, telephone, mail, email | All participants were invited via e-mail (by LW) and in case of no response, contacted by telephone. |
| 12. Sample size | How many participants were in the study? | 29 |
| 13. Non-participation | How many people refused to participate or dropped out? Reasons? | All twenty members of the programme group since 2010 were invited to participate in the study; seventeen of them agreed to participate in an interview. Reasons for non-participation were feeling that they couldn’t contribute because of being involved for only a short period, not being able to remember much and personal circumstances.  Eleven community workers working at the operational level were asked to participate in an interview; eight of them agreed to participate in an interview. Reasons for non-participation were emigration and feeling that they couldn’t contribute.  Six municipal executive councilors who have been involved in the approach since the start in 2010 and work at the strategic level, were asked to participate in an interview; five of them agreed to participate. Reason for non-participation is unknown. |
| *Setting* |  |  |
| 14. Setting of data collection | Where was the data collected? e.g. home, clinic, workplace | 27 participants were interviewed by LW (PhD candidate) at their workplace. Two participants were interviewed in a restaurant. |
| 15. Presence of non-participants | Was anyone else present besides the participants and researchers? | Other guests were present at the two interviews in the restaurant, sitting at other tables. There was no interaction with others during the interview. |
| 16. Description of sample | What are the important characteristics of the sample? e.g. demographic data, date | See table 1. |
| *Data collection* |  |  |
| 17. Interview guide | Were questions, prompts, guides provided by the authors? Was it pilot tested? | Semi-structured interview guide, see supporting information file 2. |
| 18. Repeat interviews | Were repeat interviews carried out? If yes, how many? | No |
| 19. Audio/visual recording | Did the research use audio or visual recording to collect the data? | The interviews were audio recorded and transcribed verbatim anonymously. |
| 20. Field notes | Were ﬁeld notes made during and/or after the interview or focus group? | Field notes were made during the interviews. After every interview the main conclusions and remarkable information was written in a logbook. |
| 21. Duration | What was the duration of the interviews? | The duration of the interviews ranged from 49 to 92 minutes. |
| 22. Data saturation | Was data saturation discussed? | The research team discussed findings and data saturation multiple times. |
| 23. Transcripts returned | Were transcripts returned to participants for comment and/or correction? | No |
| **Domain 3: analysis and ﬁndings** | |  |
| *Data analysis* |  |  |
| 24. Number of data coders | How many data coders coded the data? | Two: LW and DW |
| 25. Description of the coding tree | Did authors provide a description of the coding tree? | No |
| 26. Derivation of themes | Were themes identiﬁed in advance or derived from the data? | Themes were derived from data. |
| 27. Software | What software, if applicable, was used to manage the data? | MAXQDA, version 2018. |
| 28. Participant checking | Did participants provide feedback on the ﬁndings? | After analyzing the data of the semi-structured interviews, 7 of the 29 interviewed professionals participated in a verifying focus group discussion. Participants were asked to reflect on key elements that were distracted from analysis of the semi-structured interviews. The participants agreed with the preliminary conclusions drawn and only some small adjustments were made afterwards, to align the conclusions more with the experiences of the participants Adjustments did not concern the content of the conclusions, only structure and language. |
| *Reporting* |  |  |
| 29. Quotations presented | Were participant quotations presented to illustrate the themes/ﬁndings? Was each quotation identiﬁed? e.g. participant number | Yes |
| 30. Data and ﬁndings consistent | Was there consistency between the data presented and the ﬁndings? | Yes |
| 31. Clarity of major themes | Were major themes clearly presented in the ﬁndings? | Yes, see table 2 in the article. |
| 32. Clarity of minor themes | Is there a description of diverse cases or discussion of minor themes? | Yes |
