# Supplementary material for: Key elements of a successful integrated community-based approach aimed at reducing socioeconomic health inequalities in the Netherlands: A qualitative study
Source: PLoS One. 2020 Oct 20;15(10):e0240757. doi: 10.1371/journal.pone.0240757 (PMC7575081; doi:10.1371/journal.pone.0240757)
Supplement: S2 File — (DOCX) [file pone.0240757.s002.docx]

**S2 file. Interview guide - Key elements of a successful local integrated community-based approach**

| **Topic** | **Interview questions** | **Optional sub questions** |
| --- | --- | --- |
| Experience | What do you understand by the approach ZHC*? |  |
|  | What do you think is the greatest success of ZHC? | Why? What has been important in this? What was needed for that? What have you contributed yourself? What have others contributed? |
|  | Are you satisfied (except for that) about what has been achieved in the context of ZHC? | What are you satisfied with? (goals or processes or both) What has been important in this regard? What was needed for that? What have you contributed yourself? What have others contributed? |
| Local structure and coordination | What is your occupation? | Which organization? Which period? Approximately how many hours per week did you work on the ZHC approach? |
|  | Which pillar do you find most successful / strongest here locally in Zwolle? |  |
|  | Do you find the local structure of ZHC successful? If so, why? |  |
|  | Are you satisfied with the motivation and contribution of the local network (program group) to achieve the objectives? | What were the obstacles in that regard? + how solved? What were the success factors? Suggestions for improvement? |
|  | Are you satisfied with the composition of the local network (program group)? | What were the obstacles in that regard? + how solved? What were the success factors? Suggestions for improvement? |
|  | How is communication / coordination between the organizations in the local network (program group) going? | What were the obstacles in that regard? + how solved? What were the success factors? Suggestions for improvement? |
|  | How is the communication / coordination about ZHC within your own organization? E.g. with neighbourhood professionals, administrators |  |
| Support | To what extent do you think ZHC is supported by the local government? I.e. on a strategic level: the Municipal Executive and Municipal Council | What has that support been? What has been done specifically to gain that support? What were the obstacles in that regard? + how solved? What were the success factors? Suggestions for improvement? |
|  | Is there a visible ambassador from this local administration? | If so, who and what was his / her position? Did he / she actively promote the message of a healthy lifestyle? How? Is that important to you? Why? At what moments and how was he / she in the picture? Suggestions for improvement? |
|  | In what way have other policy sectors (other than health) of the local government been involved in ZHC? For example youth, education, sports, spatial planning | What did they contribute to the approach? Is that important to you? Why? At what moments and how are / were the other policy sectors involved? Was there a cross sectoral budget for ZHC? Suggestions for improvement? |
|  | To what extent is ZHC supported by the board / management of your own organization? |  |
| Participation | From 2010, children aged 019 (+ parents) were the target group of ZHC. Since 2016, seniors living at home have also been a target group.12. What do you think about the broadening? |  |
|  | What has been done to reach those target groups? | What were the obstacles in that regard? + how solved? What were the success factors? Suggestions for improvement? |
|  | Has a target group / community analysis been done at the start of ZHC? I.e. specify and explore the target groups |  |
|  | In what way have 1) children 2) parents 3) seniors participated (other than just as a participant) in the approach / activities? | Was this during the design, implementation or evaluation? Nonparticipation> informed> consulted> participation> cooperation? Do you think this is important? What were the obstacles in that regard? + how solved? What were the success factors? Suggestions for improvement? |
| Collaboration | In what way has there been collaboration with public organizations in the context of ZHC? | How did you experience this collaboration? Is the cooperation officially laid down in an agreement or contract? What has this cooperation yielded? both for ZHC and for organization. What has been done in concrete terms to bring about this cooperation? What were the obstacles in that regard? How solved? Suggestions for improvement? |
|  | What were your expectations of public-private partnerships? | To what extent have these expectations come true? |
|  | In what way has there been collaboration with private organizations in the context of ZHC? | Is the partnership formalized in an agreement or contract? How did you experience collaboration with private parties? What has this cooperation yielded? both for ZHC and for organization What has been done in concrete terms to bring about this cooperation? What were the obstacles in that regard? How solved? Suggestions for improvement? |
| Interventions and activities | Are you satisfied with the way in which interventions and activities of ZHC come about? | What was the role of the program group in this? What were the obstacles in establishing activities? + how solved? What were the success factors? Suggestions for improvement? |
|  | When do you think an intervention or activity change is successful? |  |
|  | A goal of ZHC is to make healthy choices easier in all places where citizens go. Home, school, childcare, neighbourhood, sports & leisure, media, workplace. Can you indicate per area to what extent, according to your insight, efforts have been made in the 2010-2018 period? | Do you think efforts in these environments are equally important? |
|  | Do you think that interventions and activities make sufficient use of existing knowledge at knowledge institutes or partner organizations? |  |
|  | How has the program group communicated about these interventions and activities? | What tools have been developed by the program group for communicating with the target groups? (posters, books, manuals) Has existing tools been used? Existing knowledge about communication? Who was responsible for communication from the program group? Partners too? |
| Integrated pathways prevention and care | Are you satisfied with how the connection between prevention and care is organized? Do you think healthcare is sufficiently involved? | What were the obstacles in that regard? + how solved? What were the success factors? Suggestions for improvement? |
|  | Did the transitions in the social domain in 2015 lead to changes or shifts in organizations / professionals who have the task of identifying and referring overweight children? |  |
| Monitoring and evaluation | Has ZHC been evaluated as an approach at the effect level? | How often? When? What has the evaluation yielded? Has anything been done with that? What were the obstacles in that regard? + how solved? Suggestions for improvement? |
|  | Has ZHC been evaluated as an approach at process level? | How often? When? What has the evaluation yielded? Have lessons been learned? Has anything been done with that? What were the obstacles in that regard? + how solved? Suggestions for improvement? |
|  | Have experts been designated who are responsible for evaluation? | Who? |
|  | Was there a budget for evaluation? | Where do these resources come from? |

**ZHC = Zwolle Healthy City*
