# Supplementary material for: Key elements of a successful integrated community-based approach aimed at reducing socioeconomic health inequalities in the Netherlands: A qualitative study
Source: PLoS One. 2020 Oct 20;15(10):e0240757. doi: 10.1371/journal.pone.0240757 (PMC7575081; doi:10.1371/journal.pone.0240757)
Supplement: S3 File — (DOCX) [file pone.0240757.s003.docx]

**S3 file. Examples from practice to inspire, derived from the interviews**

| **Key element** |  |
| --- | --- |
| Collaboration between a variety of local organizations | The ‘Streetmoves’ project, where professionals working at the welfare organization, regional public health authority and municipal sport services (together with citizens) organize a yearly community event with the focus on sports. By collaborating with professionals from different organizations, more resources and more expertise can be deployed in a common project. |
| Support on three levels: strategic, tactical, operational | To show the broad support at the strategic level, the approach started in 2010 with all the municipal executive councilors and representatives of organizations involved signing the approach’s plans. |
| Support on three levels: strategic, tactical, operational | To close the experienced gap between the program group at the tactic level and professionals at the operational level, intermediary professionals, called ‘health brokers’, have been appointed to bring the approach to the attention of professionals at the operational level. |
| Communication and coordination | Appointed internal coordinators who work at the involved organizations are given time to implement the approach in their organization and focus on promoting healthy lifestyle. They are also trained in the theme. |
| Collaboration with private organizations | Two interactive football walls (‘Sutu walls’) are initiated and financed by local private organizations with the aim to improve the quality of life in the neighborhood. |
| Collaboration with private organizations | Families were invited by the local supermarket for a tour that shows healthy and affordable options |
| Collaboration with citizens | Club2Move, a community sports club where youngsters can exercise twice a week and participate in activities around healthy nutrition. The youngsters participate in promotion, organization and execution of the activities. |
| Profiling the approach like a brand | Involved organizations are stimulated to place beach flags with the ZHC logo at activities to show that the activity is part of the approach. |
| Move along with, and take advantage of, (local and national) opportunities | A project in primary schools about the importance of a healthy lifestyle ("IkLekkerFit" in Dutch) was temporarily funded by the health insurance company and the local government. As the project ended, there were national financial impulses for the healthy school approach, which ensured that the investments in the earlier project could be continued and embedded. |
| Embeddedness of the approach in organization’s policy and processes | Previously, at activities organized by the welfare organization, soft drinks and crisps were self-evidently sold. This has diminished over the years. |
| Continuous monitoring and evaluating goals and processes, and learning from the results | The ChecKid study, with surveys among children and parents about dietary behavior and physical activity, and in which over 10,000 children in the municipality were weighed and measured enabled the approach to show results. |
